# Percutaneous treatment of complex biliary stone disease using endourological technique and literature review

**DOI:** 10.1590/S1679-45082015RC2935

**Published:** 2015

**Authors:** Fernando Korkes, Ariê Carneiro, Felipe Nasser, Breno Boueri Affonso, Francisco Leonardo Galastri, Marcos Belotto de Oliveira, Antônio Luiz de Vasconcellos Macedo

**Affiliations:** 1 Faculdade de Medicina do ABC, Santo André, SP, Brazil.; 2 Hospital Israelita Albert Einstein, São Paulo, SP, Brazil.; 3 Santa Casa de Misericórdia de São Paulo, São Paulo, SP, Brazil.

**Keywords:** Ureteroscopy, Choledocholithiasis, Billary tract surgical procedures, Case reports

## Abstract

Most biliary stone diseases need to be treated surgically. However, in special cases that traditional biliary tract endoscopic access is not allowed, a multidisciplinary approach using hybrid technique with urologic instrumental constitute a treatment option. We report a case of a patient with complex intrahepatic stones who previously underwent unsuccessful conventional approaches, and who symptoms resolved after treatment with hybrid technique using an endourologic technology. We conducted an extensive literature review until October 2012 of manuscripts indexed in PubMed on the treatment of complex gallstones with hybrid technique. The multidisciplinary approach with hybrid technique using endourologic instrumental represents a safe and effective treatment option for patients with complex biliary stone who cannot conduct treatment with conventional methods.

## INTRODUCTION

Most of biliary stone diseases need some type of surgical intervention. In the current scenario, the conventional treatments for this disease counts with the support of videolaparoscopy, endoscopic retrograde cholangiopancreatography (ERCP), percutaneous transhepatic cholangiography (PTC), and open surgery.^(1)^ The method to chosen depends on patient’s clinical conditions, morphological characteristics of the calculus, and specific local of the calculus in the biliary tree.

Laparoscopic cholecystectomy is established as the standard treatment for cholecystectomy. However, management of calculus in biliary tract still a challenge, mainly in complex cases and when conventional methods fail.^(2)^ Currently, most of calculus in the extrahepatic biliary tract are treated concomitantly with cholecystectomy by the anterograde exploration of common and choledochal bile ducts by the use of choledocoscopy or radiologic techniques.^(3,4)^

ERCP with papillotomy is considered gold standard for choledocolitiasis,^(5)^ particularly for its success rate of 90%.^(6)^ However, this treatment is not applicable to all cases. Some factors can limit the conduction and success of the procedure, such as large calculi, unusual localization, anatomical change of duodenal large papilla, condition after liver transplantation or previous surgeries involving the stomach with the need of Roux-en-Y derivation. For this reason, the development of alternative methods is required.

Despite the high rates of resolution, open surgery constitutes a difficult technique and presents high morbidity.^(7)^ These factors prevent this technique use in several cases.

In the beginning of 1980, because of the appearance and improvement of endoscopic materials, percutaneous nephrolithotomy and the ureteroscopy became an important alternative for treatment of renal and ureteral calculi. Because of technical progress and enhancements achieved by surgeons and interventionists to perform the procedure, the multidisciplinary approach of patients enabled a widely use of these modalities of treatment in different medical situations, such as in cases of biliary stone diseases.^(1)^

The technological development in the last years enabled the appearance of flexible and fine-gauge devices and new sources of energy for fragmentation of these calculi that, associated with knowledge of urological endoscopy and interventional radiology, is presented as a prominent alternative in the attempt to solve complex causes of biliary stone or also in cases that conventional methods have failed.^(2,8-13)^

Despite applying urologic endoscopy techniques for treatment of biliary calculus,^(14-19)^ its indications remain controversial. We report the case of a patient with complex biliary stone who underwent hybrid treatment at *Hospital Israelita Albert Einstein* (SP), Brazil, associated with the use of flexible ureteroscopy, *holmium*: *yttrium aluminumgarnet laser* (Ho:YAG) and techniques of interventional radiology for removal of calculus.

## CASE REPORT

We report a case of 47-year-old men in post-operative period of right hepatectomy conducted 6 years ago in consequence of hydatid cyst of 22cm diameter that involved large proportion of his right lobe of liver and did not respond adequately to clinical treatment. After 2 years of the procedure, the patient began to show intermittent episodes of cholangitis and consecutive stenosis of common liver duct. A retograde cholangioplasty endoscopy was conducted with implant of transpapillary biliary prostheses without success to solve the mentioned stenosis. After new recurrences of cholangitis episodes due to the use of biliary prosthesis, the patient was submitted two years ago to biliodigestive in Roux-en-Y derivation with anastomosis in hilar plate region of left lobe of the liver. In the follow-up, he progressed with new stenosis, bibliodigestive anastomosis and posterior formation of intra-hepatic calculi.

One year before the patient had undergone subxiphoid transparietal puncture on the left biliary tract followed by transparietal cholangiography that confirmed stenosis of biliodisgestive anastomosis and diagnosed the presence of diverticulum in left biliary ducts, which presented a biliary calculus measuring approximately 1cm diameter in its interior. We conducted a transparietal cholangioplasty of biliodisgetive anastomosis with cutting-balloon 7x20mm and repetitive attempts, without success, for removal of the biliary calculus using radioscopy with support of balloon catheter, guide wires and Basket catheter. We opted to implant the 8.5F internal/external transparietal biliary drainage to discuss later on the definitive management.

**Figure 1:**
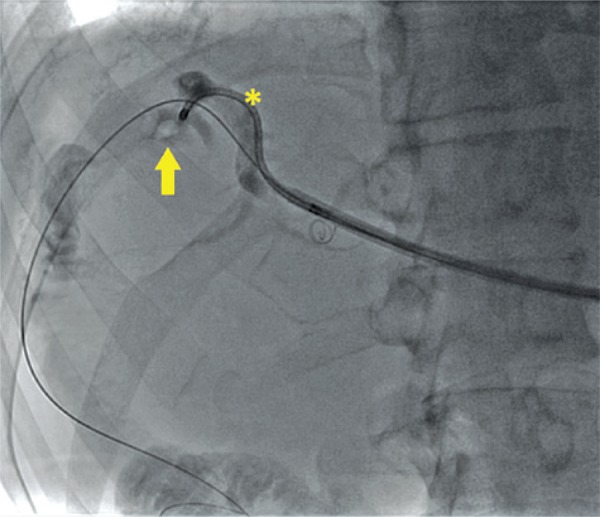
Radioscopic imaging by the flexible ureteroscopy

After 4 weeks, the previous implanted drainage was removed and the sheath 8F was implanted for the flexible ureteroscopy access. The procedure was done under general anesthesia at a hybrid room with access to fluoroscopy. An urologist along with interventionist radiologists conducted the procedure. After initial dilation, the sheath of ureteral access sheat was positioned under radioscopic control up to the point of interest (Figure 1). Biliary tract was accessed using a flexible ureteroscopy (Storz Flex-X^2^ KARL STORZ^®^) ureteroscopy until the direct visualization of the calculus. We used 0.9% sodium chloride solution for irrigation. For fragmentation of biliary calculus we used 200μm laser fiber with 0.7J/pulse of potency and 7Hz of frequency, and posteriorly fragments was removal with nitinol basket (DIMENSION^®^ Bard^®^) (Figure 1). Control cholangiography showed the treatment of lesion and lack of fragments of intra-hepatic biliary calculi. The procedure was done without intercurrences and the patient was discharged in the second day after surgery.

## DISCUSSION

Technology and technical improvements in the treatment of kidney stone has been used as an increment in management of biliary tract stone in complex cases when traditional procedures have failed.

The PTC can be done by radiologic exploration of the biliary tracts (REBT) with the removal of calculus with basket under fluoroscopy guidance, or by exploration with direct visualization using coledoscopy, ureteroscopy or nephroscopy.

The REBT, in addition of not enabling direct visualization of the calculus, cannot access calculus using unusual topographies so that increasing the chance of treatment failure.

The cholangioscopy conducted with choledoscopy presents some limitations, such as fragility, canal to access of fine gauge and little curvature, which unable its use in some cases. Hence, the use of urological technology becomes a prominent alternative in complex cases, enabling better visualization, fragmentation and removal of calculi. Some authors have reported in the literature this approach for the treatment of complex biliary stone with high rates of success and low morbidity (Chart 1).

**Chart 1:** Medical literature reports of percutaneous treatment of complex biliary stone using urologic techniques and equipment

Author	Year	n	Treated disease	Indication	Equipment used	Energy source	Success	Complications
Hoang et al.^(20)^	2007	2	Biliodisgetive anastomosis stenosis with calculus	Failure in access ERCP	Semi-rigid ureteroscopy	Not described	100%	-
Di Pisa et al.^(21)^	2008	1	Stenosis at the choledochodocus (after liver transplantation) with calculus	Failure in ERCP	Rigid nephroscopy	Ballistic lithotripter	100%	-
Ray et al.^(7)^	2009	19	Intrahepatic biliary calculus	Failure in ERCP (n=17) Contraindication of general anestesia (n=2)	Rigid and flexible nephroscopy	laser and pneumatic lithotripsy	76% (disobstruction in 94%)	Prolonged drainage (n=1) AMI (n=1)
Healy et al.^(2)^	2009	9	Intrahepatic biiary stone, Stenosis at the choledochodocus (after liver transplantation), residual calculus VLPS post-cholecystectomy, Pos-Whipple, Cholangite sclérosante	Failure in ERCP	Flexible cystoscope and ureteroscope	Laser	100%	Prolonged drainage (n=1)
Khan et al.^(22)^	2010	80	biliary calculus	cholecystectomy during VLPS	Rigid nephroscopy	Variable	99.5%	Conversion for open surgery (n=1)
Rimon et al.^(23)^	2011	22	Intrahepatic biliary stone	Failure in ERCP (n=13) Fail in access ERCP (n=9)	Flexible ureteroscopy	Laser	82%	-

ERCP: endoscopic retrograde cholangiopancreatography; VLPS: videolaparoscopic surgery. AMI: Acute myocardial infarction.

Few centers still doing hybrid treatment and few studies on this regard have been published in the literature so far, as described in chart 1. Perhaps the greatest limitation of this technique is the need of having an experienced and well-trained multidisciplinary team, and also the high cost that this technology entails. However, in special and well indicated cases, this technique seems to be the cheapest option compared with conventional procedures considering length of hospital stay and possible complications. However, no studies have evaluated such costs so far.

An crucial point for the analysis of these published studies is that percutaneous access of biliary tracts was conducted in different ways, based on topography of the pathology described (for example: transparieto-hepatic, transparieto jejunal) and, in some cases, the procedure can be concluded successfully by using semi-rigid instrument that technically facilitates and reduces treatment cost.

In our case, the use of flexible ureteroscopy was technically easy and effective. One of the main difficulties we had was the multiplicity of biliary tree, which difficult spatial localization. The principles used both in flexible ureteroscopy and interventional radiology, such as the use of intraoperative cholangiography and fluoroscopy imaging, applied simultaneously to aid direct visualization, facilitated to overcome these barriers. The high cost and extremely fragility of these devices are important limitations to consider in relation the availability and access to this technology.

## CONCLUSION

The multidisciplinary approach associated with urologic technology is a possible and efficient method. This approach can be considered as a treatment option for specific cases of complex biliary tract stone disease.

## References

[1] Ponsky LE, Geisinger MA, Ponsky JL, Streem SB (2001). Contemporary “urologic” intervention in the pancreaticobiliary tree. Urology.

[2] Healy K, Chamsuddin A, Spivey J, Martin L, Nieh P, Ogan K (2009). Percutaneous endoscopic holmium laser lithotripsy for management of complicated biliary calculi. JSLS.

[3] Duensing RA, Williams RA, Collins JC, Wilson SE (2000). Common bile duct stone characteristics: correlation with treatment choice during laparoscopic cholecystectomy. J Gastrointest Surg.

[4] Tranter SE, Thompson MH (2002). Comparison of endoscopic sphincterotomy and laparoscopic exploration of the common bile duct. Br J Surg.

[5] Williams EJ, Green J, Beckingham I, Parks R, Martin D, Lombard M, British Society of Gastroenterology (2008). Guidelines on the management of common bile duct stones (CBDS). Gut.

[6] Neuhaus H (2003). Endoscopic and percutaneous treatment of difficult bile duct stones. Endoscopy.

[7] Ray AA, Davies ET, Duvdevani M, Razvi H, Denstedt JD (2009). The management of treatment-resistant biliary calculi using percutaneous endourologic techniques. Can J Surg.

[8] Gamal EM, Szabó A, Szüle E, Vörös A, Metzger P, Kovacs G (2001). Percutaneous video choledochoscopic treatment of retained biliary stones via dilated T-tube tract. Surg Endosc.

[9] Hazey JW, McCreary M, Guy G, Melvin WS (2007). Efficacy of percutaneous treatment of biliary tract calculi using the holmium: YAG laser. Surg Endosc.

[10] Monga M, Gabal-Shehab LL, Kamarei M, D’Agostino H (1999). Holmium laser lithotripsy of a complicated biliary calculus. J Endourol.

[11] Ogawa K, Ohkubo H, Abe W, Maekawa T (2002). Percutaneous transhepatic small-caliber choledochoscopic lithotomy: a safe and effective technique for percutaneous transhepatic common bile duct exploration in high-risk elderly patients. J Hepatobiliary Pancreat Surg.

[12] Shamamian P, Grasso M (2004). Management of complex biliary tract calculi with a holmium laser. J Gastrointest Surg.

[13] Teichman JM, Schwesinger WH, Lackner J, Cossman RM (2001). Holmium: YAG laser lithotripsy for gallstones. A preliminary report. Surg Endosc.

[14] Adamek HE, Maier M, Jakobs R, Wessbecher FR, Neuhauser T, Riemann JF (1996). Management of retained bile duct stones: a prospective open trial comparing extracorporeal and intracorporeal lithotripsy. Gastrointest Endosc.

[15] Costamagna G, Gabbrielli A, Mutignani M, Perri V, Pandolfi M, Boscaini M (1997). Extracorporeal shock wave lithotripsy of pancreatic stones in chronic pancreatitis: immediate and medium-term results. Gastrointest Endosc.

[16] Hochberger J, Bayer J, May A, Muhldorfer S, Maiss J, Hahn EG (1998). Laser lithotripsy of difficult bile duct stones: results in 60 patients using a rhodamine 6G dye laser with optical stone tissue detection system. Gut.

[17] Sauerbruch T, Holl J, Sackmann M, Werner R, Wotzka R, Paumgartner G (1987). Disintegration of a pancreatic duct stone with extracorporeal shock waves in a patient with chronic pancreatitis. Endoscopy.

[18] Stage JG, Moesgaard F, Grønvall S, Stage P, Kehlet H (1998). Percutaneous transhepatic cholelithotripsy for difficult common bile duct stones. Endoscopy.

[19] Wolf JS, Nakada SY, Aliperti G, Edmundowicz SA, Clayman RV (1995). Washington University experience with extracorporeal shock-wave lithotripsy of pancreatic duct calculi. Urology.

[20] Hoang JK, Little AF, Clarke A (2007). Percutaneous intracorporeal lithotripsy of biliary calculi. Australas Radiol.

[21] Di Pisa M, Traina M, Miraglia R, Maruzzelli L, Volpes R, Piazza S (2008). A case of biliary stones and anastomotic biliary stricture after liver transplant treated with the rendez-vous technique and electrokinetic lithotritor. World J Gastroenterol.

[22] Khan M, Qadri SJ, Nazir SS (2010). Use of rigid nephroscope for laparoscopic common bile duct exploration-a single-center experience. World J Surg.

[23] Rimon U, Kleinmann N, Bensaid P, Golan G, Garniek A, Khaitovich B (2011). Percutaneous transhepatic endoscopic holmium laser lithotripsy for intrahepatic and choledochal biliary stones. Cardiovasc Intervent Radiol.

