# Supplementary material for: Percutaneous treatment of complex biliary stone disease using endourological technique and literature review
Source: Einstein (Sao Paulo). 2015 Oct-Dec;13(4):611–5. doi: 10.1590/S1679-45082015RC2935 (PMC4878640; doi:10.1590/S1679-45082015RC2935)
Supplement: Translation

# Translation: Tratamento percutâneo da litíase biliar complexa intra-hepática com técnica endourológica e revisão da literatura

**Authors:** Fernando Korkes, Ariê Carneiro, Felipe Nasser, Breno Boueri Affonso, Francisco Leonardo Galastri, Marcos Belotto de Oliveira, Antônio Luiz de Vasconcellos Macedo

**Affiliations:** 1 Faculdade de Medicina do ABC, Santo André, SP, Brasil.; 2 Hospital Israelita Albert Einstein, São Paulo, SP, Brasil.; 3 Santa Casa de Misericórdia de São Paulo, São Paulo, SP, Brasil.

**Keywords:** Ureteroscopia, Coledocolitiase, Procedimentos cirúrgicos do sistema biliar, Relatos de casos

## Abstract

A maioria das doenças litiásicas das vias biliares necessita de tratamento cirúrgico. No entanto, em casos especiais, que não permitem o acesso tradicional das vias biliares por via endoscópica, a abordagem multidisciplinar com técnica híbrida, que utiliza instrumentais endourológicos, apresenta-se como uma opção no tratamento. Relatamos aqui o caso de um paciente com litíase de via biliar complexa com múltiplas abordagens prévias, sem sucesso no tratamento com métodos convencionais, resolvido com abordagem híbrida com tecnologia endourológica. Realizamos uma extensa busca na literatura de artigos relacionados ao tratamento de litíase biliar complexa com técnica híbrida publicados no PubMed até outubro de 2012. A abordagem multidisciplinar com técnica híbrida, utilizando instrumental endourológico, representa uma opção segura e eficaz no tratamento de pacientes com litíase complexa de via biliar com impossibilidade de tratamento com métodos convencionais.

## INTRODUÇÃO

A maioria das doenças litiásicas das vias biliares necessita de algum tipo de intervenção cirúrgica para seu tratamento. No cenário atual, o arsenal terapêutico convencional para essa doença conta com o auxílio de videolaparoscopia, colangiopancreatografia retrógrada endoscópica (CPRE), colangiografia trans-hepática percutânea (CTP) e cirurgia aberta (CA).^(1)^ A escolha do método a ser utilizado depende das condições clínicas do paciente, das características morfológicas do cálculo e da localização da árvore biliar em que ele se encontra.

A colecistectomia laparoscópica está consagrada como tratamento padrão da colelitíase. No entanto, o manejo de cálculos em vias biliares ainda consiste em um desafio, principalmente em casos complexos e na falha dos métodos convencionais.^(2)^ Atualmente, a maior parte dos cálculos situados nas vias biliares extra-hepáticas são tratados concomitantes à colecistectomia, por meio da exploração anterógrada do ducto hepático comum e colédoco, com auxílio do coledocoscópio ou técnicas radiológicas.^(3, 4)^

A CPRE com papilotomia é considerada o tratamento de primeira linha para coledocolitíase,^(5)^ já que possui taxa de sucesso de 90%.^(6)^ No entanto, não é possível de ser realizada em todos os casos. Fatores como cálculos volumosos, localização atípica, alteração anatômica da papila de duodenal maior, presença de divertículo duodenal, condições pós-transplante hepático ou cirurgias prévias que envolvam o estômago, com necessidade de derivação em Y de Roux, são limitantes para a realização e o sucesso desse procedimento, daí a necessidade de desenvolvimento de métodos alternativos.

A CA, apesar dos altos índices de resolução, é acompanhada de grande dificuldade técnica e morbidade.^(7)^ Isso inviabiliza, em muitos casos, seu uso.

No início da década de 1980, com o surgimento e o aperfeiçoamento de materiais endoscópicos, a nefrolitotripsia percutânea e a ureteroscopia surgiram como uma importante alternativa para o tratamento do cálculo renal e ureteral. Em consonância com essa evolução e com o aprimoramento técnico dos cirurgiões e intervencionistas, a abordagem multidisciplinar dos pacientes possibilitou que essas modalidades de tratamento fossem amplamente utilizadas e aplicadas em situações médicas distintas, como nos casos de litíase das vias biliares.^(1)^

Nos últimos anos, o desenvolvimento tecnológico proporcionou o surgimento de aparelhos de fino calibre, flexíveis e de novas fontes de energia para a fragmentação desses cálculos que, associados aos conhecimentos da endoscopia urológica e da radiologia intervencionista, apresentam-se como alternativas promissoras na tentativa de solucionar casos complexos de litíase biliar ou, também, na falha ou na impossibilidade da realização dos métodos convencionais.^(2,8-13)^

Embora seja factível o emprego de técnicas da endoscopia urológica para tratamento de cálculo em vias biliares,^(14-19)^ suas indicações ainda não estão bem definidas. Este relato de caso teve como objetivo descrever um tratamento híbrido realizado no Hospital Israelita Albert Einstein, localizado na cidade de São Paulo (SP), Brasil, de um paciente com colédoco litíase complexa, que associou o uso do ureteroscópio flexível,* holmium*: *yttrium aluminumgarnet laser* (Ho:YAG) e técnicas de radiologia intervencionista para remoção do cálculo.

## RELATO DE CASO

Paciente de 47 anos, sexo masculino, em pós-operatório de hepatectomia direita realizada havia 6 anos devido cisto hidático de 22cm de diâmetro, que ocupava grandes proporções do lobo hepático direito e não respondera ao tratamento clínico adequado. Evoluiu, após 2 anos do procedimento, com episódios intermitentes de colangite e consecutiva estenose do ducto hepático comum. Foi realizada colangioplastia endoscópica retrógrada com implante de próteses biliares transpapilares, sem sucesso na resolução da estenose referida. Havia 2 anos, após novas recidivas de episódios de colangite, a despeito do uso das próteses biliares, fora submetido à derivação biliodigestiva em Y de Roux, com anastomose na região da placa hilar do lobo hepático esquerdo. No seguimento, evoluiu com nova estenose, agora da anastomose biliodigestiva e posterior formação de cálculos intra-hepáticos.

Havia 1 ano, fora realizada punção transparietal subxifoídea da via biliar esquerda, seguida de colangiografia transparietal, que confirmou a estenose da anastomose biliodigestiva e diagnosticou a presença de divertículo em ducto biliar esquerdo, o qual abrigava cálculo biliar de aproximadamente 1cm de diâmetro em seu interior. Realizada colangioplastia trasparietal da anastomose biliodigestiva com Cutting-Balloon 7x20mm e tentativas repetidas, sem sucesso, de exérese do cálculo biliar por meio de radioscopia com o auxílio de cateteres-balão, fios-guia e cateteres do tipo Basket. Foi feita opção pelo implante de dreno biliar transparietal interno/externo 8,5F, para posterior discussão da conduta definitiva.

Após 4 semanas, foi retirado o dreno previamente implantado e realizado implante de introdutor 8F para acesso do ureteroscópio flexível. O procedimento foi realizado sob anestesia geral, em sala híbrida, com acesso à fluoroscopia, por médico urologista, em conjunto com radiologistas intervencionistas. Após dilatação inicial, foi locada a bainha de acesso ureteral sob controle radioscópico até o ponto de interesse (Figura 1). A via biliar foi acessada com ureteroscópio flexível (Storz Flex-X^2^ KARL STORZ^®^), até a visualização direta do cálculo, sendo utilizada solução de cloreto de sódio 0,9% para irrigação. Foi realizada a utilização de fibra laser 200μm, com potência de 0,7J/pulso e frequência de 7Hz, para fragmentação do cálculo biliar e posterior retirada dos fragmentos com basket de nitinol (DIMENSION^®^ Bard^®^) (Figura 1). A colangiografia de controle mostrou o tratamento da lesão e a ausência de fragmentos de cálculos biliares intra-hepáticos. O procedimento transcorreu sem intercorrências e o paciente recebeu alta no segundo dia pós-operatório.

**Figura 1:** Imagem radioscópica do procedimento com o auxílio do ureteroscópio flexível

## DISCUSSÃO

A tecnologia e o aprimoramento técnico oriundos do tratamento de litíase urinária têm sido utilizados como incremento no tratamento de litíase de vias biliares em casos complexos, na falha ou impossibilidade de realização dos procedimentos tradicionais.

O tratamento TPH pode se dar pela exploração radiológica das vias biliares (ERVB), com retirada do cálculo com basket guiado por fluoroscopia, ou por meio de exploração com visualização direta com utilização de coledoscópio, ureteroscópio ou nefroscópio.

A ERVB, além de não permitir visualização direta do cálculo, não consegue acessar cálculos em topografias não habituais, aumentando, assim, a chance de falha no tratamento.

A colangioscopia realizada com coledocoscópio apresenta algumas limitações, como fragilidade, canal de trabalho de fino calibre e pouca curvatura, inviabilizando seu uso em alguns casos. Assim, o uso de tecnologia urológica passa a ser uma alternativa promissora em casos complexos, permitindo melhor visualização, fragmentação e retirada dos cálculos. Dessa forma, alguns autores têm reportado, na literatura, essa forma de abordagem para o tratamento de casos complexos de litíase biliar, com altos índices de sucesso associados à baixa morbidade (Quadro 1).

**Quadro 1:** Relatos na literatura médica de tratamento percutâneo de litíase biliar complexa utilizando técnicas e equipamentos urológicos

Autor	Ano	n	Patologia tratada	Indicação	Equipamento utilizado	Fonte de energia	Sucesso	Complicações
Hoang et al.^(20)^	2007	2	Estenose de anastomose biliodigestiva com cálculo	Falha de acesso para CPRE	Ureteroscópio semirrígido	Não descrita	100%	-
Di Pisa et al.^(21)^	2008	1	Estenose de anastomose colédoco-colédoco (pós-transplante hepático) com cálculo	Falha da CPRE	Nefroscópio rígido	Litotritor balístico	100%	-
Ray et al.^(7)^	2009	19	Cálculo via biliar intra-hepatica	Falha da CPRE (n=17) Contraindicação de anestesia geral (n=2)	Nefroscópio rígido e flexível	Litotridor pneumático e laser	76% (desobstrução em 94%)	Drenagem prolongada (n=1) IAM (n=1)
Healy et al.^(2)^	2009	9	Litíase via biliar intra-hepática, estenose da anastomose colédoco-colédoco pós-transpalnte hepático, cálculo residual pós-colecistectomia VLP, pós-Whipple, colangite esclerosante	Falha da CPRE	Cistoscópio e ureteroscópio flexível	Laser	100%	Drenagem prolongada (n=1)
Khan et al.^(22)^	2010	80	Cálculo via biliar	Coledocolitiase durante VLP	Nefroscópio rígido	Variada	99,5%	Conversão para cirurgia aberta (n=1)
Rimon et al.^(23)^	2011	22	Litíase biliar intra-hepática	Falha da CPRE (n=13) Falha de acesso para CPRE (n=9)	Ureteroscópio flexível	Laser	82%	-

CPRE: colangiopancreatografia retrógrada endoscópica; VLP: videolaparoscopia; IAM: infarto aguda do miocárdio.

Como visto no quadro 1 , ainda são poucos os centros que realizam o tratamento híbrido e existem poucos estudos publicados na literatura. Talvez o grande limitante dessa técnica seja a necessidade de ter uma equipe multidisciplinar experiente e bem treinada, além do custo que essa tecnologia demanda. No entanto, em casos especiais e bem indicados, parece ser uma opção ainda mais barata que os procedimentos convencionais, quando coloca-se em questão o tempo de internação e as complicações. No entanto, ainda não há estudos que avaliem esses custos.

Um ponto importante na análise dos estudos da literatura é que o acesso percutâneo das vias biliares foi realizado de diferentes formas, de acordo com a topografia da patologia em questão (por exemplo: transparieto-hepática, transparieto jejunal), e, em alguns casos, o procedimento pode ser concluído com sucesso com utilização de instrumental semirrígido, o que facilita tecnicamente e diminui o custo do tratamento.

No presente caso, a utilização do ureteroscópio flexível mostrou-se tecnicamente fácil e efetiva. Uma das principais dificuldades encontradas foi a multiplicidade da árvore biliar, o que dificultou a localização espacial. Contudo, princípios utilizados tanto em ureteroscopia flexível como na radiologia intervencionista, tais como a utilização de colangiografia intraoperatória e a imagem fluoroscópica, simultâneas à visualização direta, facilitaram a superação desses obstáculos. Adicionalmente, o elevado custo e a fragilidade extrema desses aparelhos são limitantes importantes da disponibilidade e do acesso a essa tecnologia.

## CONCLUSÃO

A abordagem multidisciplinar com utilização de tecnologia urológica é um método factível e eficaz, podendo ser considerada uma opção para tratamento de casos específicos de litíase de vias biliares complexa.
